# FCRL3 Gene Polymorphisms Confer Autoimmunity Risk for Allergic Rhinitis in a Chinese Han Population

**DOI:** 10.1371/journal.pone.0116419

**Published:** 2015-01-16

**Authors:** Zheng Gu, Su-Ling Hong, Xia Ke, Yang Shen, Xiao-Qiang Wang, Di Hu, Guo-Hua Hu, Hou-Yong Kang

**Affiliations:** Department of Otorhinolaryngology, The First Affiliated Hospital of Chongqing Medical University, Yuzhong District, Chongqing, PR China; University of Birmingham, UNITED KINGDOM

## Abstract

**Background:**

Heredity and environmental exposures may contribute to a predisposition to allergic rhinitis (AR). Autoimmunity may also involve into this pathologic process. FCRL3 (Fc receptor-like 3 gene), a novel immunoregulatory gene, has recently been reported to play a role in autoimmune diseases.

**Objective:**

This study was performed to evaluate the potential association of FCRL3 polymorphisms with AR in a Chinese Han population.

**Methods:**

Five single-nucleotide polymorphisms of FCRL3, rs945635, rs3761959, rs7522061, rs10489678 and rs7528684 were genotyped in 540 AR patients and 600 healthy controls using a PCR-restriction fragment length polymorphism assay. Allele, genotype and haplotype frequencies were compared between patients and controls using the χ2 test. The online software platform SHEsis was used to analyze their haplotypes.

**Results:**

This study identified three strong risk SNPs rs7528684, rs10489678, rs7522061 and one weak risk SNP rs945635 of FCRL3 in Chinese Han AR patients. For rs7528684, a significantly increased prevalence of the AA genotype and A allele in AR patients was recorded. The frequency of the GG genotype and G allele of rs10489678 was markedly higher in AR patients than those in controls. For rs7522061, a higher frequency of the TT genotype, and a lower frequency of the CT genotype were found in AR patients. Concerning rs945635, a lower frequency of the CC genotype, and a higher frequency of G allele were observed in AR patients. According to the analysis of the three strong positive SNPs, the haplotype of AGT increased significantly in AR cases (AR = 38.8%, Controls = 24.3%, P = 8.29×10-14, OR [95% CI] 1.978 [1.652~2.368]).

**Conclusions:**

This study found a significant association between the SNPs in FCRL3 gene and AR in Chinese Han patients. The results suggest these gene polymorphisms might be the autoimmunity risk for AR.

## Introduction

Allergic rhinitis (AR) is defined as an IgE-mediated nasal inflammation, which is induced by allergens and regulated by T lymphocytes [1]. AR has a major impact on quality of life by causing unpleasant symptoms such as sneezing, nasal congestion, nasal itching, rhinorrhea and the obstruction of the nasal passages. Furthermore, AR is a known risk factor for comorbid conditions such as asthma, rhinosinusitis, nasal polyposis, and sleep disorders, resulting in important medical and social problems [1–4]. This disease has an estimated worldwide incidence rate of 10–20% [5]. Recent decades, the prevalence of AR has risen sharply [6]. Our group has investigated the epidemiology of AR in western China (Poor developed area in China), the prevalence of self-reported AR was 32.30% (Chongqing), 34.3% (Chengdu), 37.9% (Urumqi), and 30.3% (Nanning) [7]. Although its etiology and pathogenesis are not completely understood, accumulating evidence shows that environmental and heredity exposures may contribute to a predisposition to AR. Over the last two decades, the pathogenesis of AR has been widely studied and genetic factors are thought to be major players affecting the development, severity and treatment of AR [8, 9].

It has been reported that numerous loci and candidate genes show associations with AR [8, 9]. In our previous research work, we have reported the association between Interleukin-23R gene [10], interleukin-12 receptor β1/β2 genes [11], Interleukin-27 gene [12] polymorphisms and susceptibility to AR in Chinese population. These results show a debate about the imbalance between Th1 and Th2 cells in relation with the pathogenic mechanisms of AR. However, the details of the underlying pathogenic mechanisms remain unclear. Recently, another research group [13–17] at our lab has investigated the association of Behçet’s disease with multiple immune response genes and has identified multiple Behçet’s disease-related immunoregulatory pathways in the Chinese Han population. A large number of gene polymorphisms, including FCRL3 (Fc receptor-like 3) gene were identified significant associations with Behçet’s disease. Since these genetic predisposition studies support an important role for lymphocyte differentiation as well as ubiquitination pathways, we postulated the FCRL3 gene may involve in the pathogenic process of AR.

Fc receptor-like genes (FCRLs), also known as FCRHs (Fc receptor homology), locate at the 1q21–23 region [18]. FCRLs have the similarity in structure and sequence to the classical Fcγ receptor genes (FcγR). They contain six immunoglobulin (Ig) superfamily members, FCRL1–FCRL6, according to their chromosomal order [19]. Among of these genes, FCRL3 is predominantly expressed in lymphoid organs, more precisely in germinal centers, and has been linked to the maturation of B cells [20]. It contains tyrosine-based activation and inhibition motifs in its cytoplasmic domain, suggesting that it plays a role in immune cell regulation [21]. FCRL3 may involve in the process of the differentiation of B cells into autoreactive cells and has been presumed to function through modulating signal transduction via activation/inactivation of signaling tyrosine protein kinases [21].

Recently, polymorphisms of FCRL3 have been reported to be associated with rheumatoid arthritis (RA), systemic lupus erythematosus (SLE) [22], Behcet’s disease [23], autoimmune thyroid disease (AITD) [22, 24], and multiple sclerosis (MS) [25, 26]. As an allergic disease which also is mediated by B- and T-cell response, AR may share a similar pathogenesis. Whether SNPs of FCRL3 are also associated with the susceptibility to AR is not yet known, therefore, we conducted a case—control study and analyzed the SNPs within FCRL3. In accordance with our aim, five SNPs of FCRL3, including rs945635, rs3761959, rs7522061, rs10489678 and rs7528684, were genotyped in 540 AR patients and 600 healthy controls using a PCR-restriction fragment length polymorphism assay (PCR-RFLP). This study identified three strong risk SNP rs7528684, rs10489678, rs7522061 and one weak risk SNPs rs945635 of FCRL3 in Chinese Han AR patients. The online software platform SHEsis was used to analyze the haplotypes of above chosen five loci SNPs.

## Methods

### Study population

Five hundred and forty AR patients referred to us from January 2012 to July 2013 were included in this study. All the patients were of Chinese Han ethnic origin, and all were from the Chongqing city and its neighboring townships in the southwest region of China. All the patients were from and had been treated at the outpatient department of Otorhinolaryngology of the First Affiliated Hospital of Chongqing Medical University, Chongqing, China. The diagnosis of AR was based on the patients’ medical history, symptoms and the presence of a positive skin prick test (SPT, Allergopharma, Hamburg, Germany) in response to a panel of common allergens defined by the ARIA 2008 guidelines [27]. The SPT results were diagnosed in accordance with the recommendations of the Subcommittee on Allergen Standardization and Skin Tests of the European Academy of Allergy and Clinical Immunology [28]. A positive SPT result was defined as the formation of a wheal larger than or equal to one half the diameter of the histamine control wheal, and at least 3 mm larger than the diameter of the negative control wheal. A total of 18 inhaled allergens were tested, including house dust, grass, tree, mold, food, cat and dog dander. In contrast, patients with accompanying systemic disease were excluded from the study. 600 healthy volunteers of the same ethnicity as the patients were recruited as the control group. The clinical characteristics of the AR patients and healthy controls were assessed at the time of diagnosis and are summarized in Table 1.

**Table 1:** Clinical features and demographic characteristics in AR patients and healthy controls.

**Characteristic**	**AR**	**Control**	***P* values**
Gender (male/female)	281/259	305/295	NS
Age (mean ± SD)	36.5±12.8	34.2±12.4	NS
Occupation (indoor/outdoor)	317/223	413/287	NS
Allergen category (%)			
House dust mite	251 (46.48)	0	<0.01
Pollens	85 (15.74)	0	<0.01
Mixed allergens	204 (37.78)	0	<0.01

AR allergic rhinitis, NS not significant

### Ethics Statement

The local ethics authorities (the Ethics Committee of the First Affiliated Hospital of Chongqing Medical University, Chongqing, China) provided permission and helped to obtain informed consent from all participants. Written informed consent was obtained from all participants.

### SNP selection and genotyping

We studied 5 SNPs rs945635, rs3761959, rs7522061, rs10489678 and rs7528684 in the FCRL3 region on 1q21–23, which were demonstrated earlier by other groups to be associated with certain immune-related diseases. SNP rs7528684 lies in the promoter region, while SNPs rs945635 and rs7522061 are located in the exon2 and exon4 region, SNPs rs3761959 and rs10489678 are located in the intron region in FCRL3 gene, respectively. Genomic DNA was extracted from EDTA-anticoagulated peripheral blood leukocytes using the Wizard Genomic DNA Purification Kit method. Briefly, 300 µl of blood was mixed with cell lysis solution. Leucocytes were spun down and lysed with Nuclei Lysis Solution. The pellet was separated out by Protein Precipitation Solution. Precipitated proteins were removed by centrifugation. Then, two-filar DNA was separated out by methyl alcohol. The DNA on the EP tube was dissolved in 100 μL DNA Rehydration Solution.

### Real Time PCR

These five SNPs were genotyped by restriction fragment length polymorphism analysis (PCR-RFLP). The amplification was performed using initial denaturation at 95°C for 5 min, 95°C for 30 s, 56–60°C for 30 s, 72°C for 30 s, and 72°C for 5 min followed by 37 cycles. The PCR products were incubated with restriction enzymes (Table 2) for at least 4 h. The primer sequences and reaction conditions used in this study are shown in Table 2. The obtained digestion products were visualized on a 4% agarose gel and stained with Gold View (SBS Genentech, Beijing, China). To confirm the genotyping results, PCR-amplified DNA samples were examined by direct sequencing (20% of all the blood samples), and the results were 100% concordant.

**Table 2:** Primers and restriction enzymes used for RFLP analysis of the FcRL3 gene.

**rs number**	**Primers**	**Tm (°C)**	**Restriction enzyme**
rs7528684	5‘ATAATTCTTTCTGTATTTTTCATATGGGAA 3’	58	FaqI
	5‘TTGTTATAAACACTGTGAAAAAAACACA3’		
rs945635	5‘TTATAGCCCATCTACTCACTCAGGATCA 3’	56	HaeIII
	5‘CCGGGATTGAGATACAAACAGCATTT3’		
rs3761959	5‘AATCAGGTAAGTTTCTCCTTCTCTCTGC 3’	60	MspI
	5‘GTGGATGGGATCTATTTCTATGTCCTTT3’		
rs7522061	5‘TCACCAGGTGAAAATTCCACATGCAC 3’	60	TaqI
	5‘TTTCTGCTCAATTTTCCACCCTGGTC3’		
rs10489678	5‘GAGAAACTTACCTGATTGTTCTCTTCCA 3’	56	Hin1II
	5‘AAGGAGGATAAACCATTTCTGTAAGTCC3’		

### Statistical analysis

All statistical analyses were performed using SPSS version 18.0 software (SPSS Inc., Chicago, Illinois, USA). *P* values of less than 0.05 were considered statistically significant. To evaluate the quality of the genotyping data, the Hardy–Weinberg equilibrium (HWE) for SNP genotype frequencies was tested using a chi-square teat (χ^2^ test). Allelic and genotypic frequencies between patients with AR and the control patients were compared by χ^2^ test. The online software platform SHEsis (http://analysis2.bio-x.cn/myanalysis.php) was used to analyze the haplotype and probabilities. The association between genotypes/alleles and the AR risk was estimated by calculating odds ratios (OR) and 95% confidence intervals (CI).

## Results

### Clinical characteristics of the study participants

The demographics of the cases and controls enrolled in this study are shown in Table 1. There were no significant differences between the cases and controls in terms of the mean age and gender distribution. 251 (46.48%) patients were sensitive to house dust mite, 85 (15.74%) were sensitive to tree pollen and 204 (37.78%) were sensitive to multiple allergens.

### Genotype distribution of the FCRL3 polymorphisms

Our results showed that FCRL3 SNPs rs945635, rs3761959, rs7522061, rs10489678 and rs7528684 were in Hardy–Weinberg equilibrium in the cases and controls (*P*>0.01). The genotype and allele frequencies of these five SNPs tested FCRL3 polymorphisms are shown in Table 3. The call rate for the examined five SNPs was 100%.

**Table 3:** Frequencies of alleles and genotypes of FCRL3 polymorphisms in AR patients and controls.

**SNP**	**Genotype**	**AR(%)**	**Control(%)**	**χ^2^**	**P value**	**Pc value**	**OR(95%CI)**
	**Allele**	**(N = 540)**	**(N = 600)**				
rs7528684	AA	249(46.1)	172(28.7)	37.13	1.10×10^-9^	1.61×10^-9^	2.13(1.67–2.72)
	AG	221(40.9)	313(52.2)	14.42	1.46×10^-4^	1.85×10^-4^	0.64(0.50–0.80)
	GG	70(13.0)	115(19.2)	8.05	4.56×10^-3^	5.86×10^-3^	0.63(0.46–0.87)
	A	719(66.6)	657(54.7)	33.21	8.27×10^-9^	1.06×10^-8^	1.65(1.39–1.95)
	G	361(33.4)	543(45.3)	12.64	8.27×10^-9^	1.06×10^-8^	0.61(0.51–0.72)
rs10489678	AA	13(2.4)	24(4.0)	2.30	0.13	0.18	0.59(0.30–1.18)
	AG	132(24.4)	210(35.0)	15.07	1.03×10^-4^	1.34×10^-4^	0.60(0.46–0.78)
	GG	395(73.1)	366(61.0)	18.90	1.38×10^-5^	1.83×10^-5^	1.74(1.36–2.24)
	A	158(14.6)	258(21.5)	17.99	2.22×10^-5^	2.83×10^-5^	0.63(0.50–0.78)
	G	922(85.4)	942(78.5)	17.99	2.22×10^-5^	2.83×10^-5^	1.60(1.29–1.99)
rs7522061	CC	80(14.8)	104(17.3)	1.33	0.25	0.28	0.83(0.60–1.14)
	CT	239(44.3)	311(51.8)	6.53	0.010	0.013	0.74(0.58–0.93)
	TT	221(40.9)	185(30.8)	12.6	3.80×10^-4^	4.81×10^-4^	1.55(1.22–1.98)
	C	399(36.9)	519(43.2)	9.40	2.17×10^-3^	2.51×10^-3^	0.77(0.65–0.91)
	T	681(63.1)	681(56.8)	9.40	2.17×10^-3^	2.51×10^-3^	1.30(1.10–1.54)
rs945635	CC	135(25.0)	182(30.3)	4.03	0.04	0.05	0.77(0.59–0.99)
	CG	289(53.5)	309(51.5)	0.46	0.50	0.53	1.08(0.86–1.37)
	GG	116(21.5)	109(18.2)	1.97	0.16	0.18	1.23(0.92–1.65)
	C	559(51.8)	673(56.1)	4.28	0.04	0.04	0.84(0.71–0.99)
	G	521(48.2)	527(43.9)	4.28	0.04	0.04	1.19(1.01–1.40)
rs3761959	AA	109(20.2)	115(19.2)	0.19	0.67	0.72	1.07(0.80–1.43)
	AG	278(51.5)	309(51.5)	3.90×10^-5^	1.00	1.00	1.00(0.79–1.26)
	GG	153(28.3)	176(29.3)	0.14	0.71	0.76	0.95(0.74–1.23)
	A	496(45.9)	539(44.9)	0.23	0.63	0.65	1.04(0.88–1.23)
	G	584(54.1)	661(55.1)	0.23	0.63	0.65	0.96(0.81–1.13)

AR, allergic rhinitis; SNP, single-nucleotide polymorphism. Pc: Corrected p value; OR: odds ratios

The results showed that there were significant differences between the AR patients and control patients concerning the frequencies of rs7522061, rs10489678 and rs7528684. A significantly increased prevalence of the rs7528684 AA genotype and A allele was found in the AR patients compared to the controls (Bonferroni corrected *P* value *(Pc)* = 1.61×10^-9^, OR [95% CI] 2.13 [1.67–2.72]; *Pc* = 1.06×10^-8^, OR [95% CI] 1.65 [1.39–1.95], respectively). The frequencies of the AG and GG genotype and the G allele were significantly lower in the AR patients than in the controls (*Pc* = 1.85×10^-4^, OR 0.64, 95% CI 0.50–0.80; *Pc* = 5.86×10^-3^, OR 0.0.63, 95% CI 0.46–0.87; *Pc* = 1.06×10^-5^, OR 0.61, 95% CI 0.51–0.72, respectively).

For rs10489678, a higher frequency of the GG genotype (*Pc* = 1.83×10^-5^, OR 1.74, 95% CI 1.36–2.24) and the G allele (*Pc* = 2.83×10^-5^, OR 1.60, 95% CI 1.29–1.99), and a lower frequency of the AG genotype (*Pc* = 1.34×10^-4^, OR 0.60, 95% CI 0.46–0.78) and the A allele (*Pc* = 2.83×10^-5^, OR 0.63, 95% CI 0.50–0.78) were found in AR patients compared with the controls.

Concerning rs7522061, a higher frequency of the TT genotype (*P*c = 4.81×10^-4^, OR [95% CI] 1.55 [1.22–1.98]) and T allele (*P*c = 2.51×10^-3^, OR [95% CI] 1.30[1.10–1.54]), and a lower frequency of the CT genotype (*P*c = 0.013, OR [95% CI] 0.74 [0.58–0.93]) and C allele (*P*c = 2.51×10^-3^, OR [95% CI] 0.77[0.65–0.91]) were found in AR patients.

For rs945635, a lower frequency of the CC genotype (*P*c = 0.05, OR [95% CI] 0.77 [0.59–0.99]) and C allele (*P*c = 0.04, OR [95% CI] 0.84 [0.71–0.99]), and a higher frequency of G allele (*P*c = 0.04, OR [95%CI] 1.19 [1.01–1.40]) were observed in AR patients.

No differences in the genotype frequency of the rs3761959 SNP were observed between the AR cohort and the control group.

### Haplotype analysis of the FCRL3 gene

Haplotype analysis was performed using the Haploview V3.32 program and the online software platform SHEsis. The 18 possible haplotype frequencies are shown in Table 4. The CGTGA, GATGA and GGTGA haplotypes, constructed by SNPs including rs945635, rs3761959, rs7522061, rs10489678 and rs7528684, accounted for about 10% in the cases, and significant higher than those in the controls *(P* = 1.52×10^-3^, OR [95% CI] 1.625 [1.201–2.918]; ). We also found that the distribution rate of the CGTGG and GGCGG haplotypes were notably decreased in the AR patients compared to the control patients (*P* = 2.36×10^-6^, OR [95% CI] 0.406 [0.277–0.597]; *P* = 3.01×10^-13^, OR [95% CI] 0.149 [0.083–0.267], respectively). Furthermore, as shown in Table 5, when only the three positive SNPs (rs7528684, rs10489678 and rs7522061) were analyzed, the haplotype of AGT increased significantly in AR cases (AR = 38.8%, the controls = 24.3%, *P* = 8.29×10^-14^, OR [95% CI] 1.978 [1.652~2.368]). On the contrary, GGC, GGT decreased markedly in AR group (AR = 11.3%, the controls = 16.4%, *P* = 4.48×10^-5^, OR [95% CI] 0.649 [0.509~0.827]; AR = 15.3%, the controls = 20.3%, *P* = 1.908×10^-5^, OR [95% CI] 0.709 [0.570~0.881], respectively).

**Table 4:** Frequencies of the haplotypes formed by rs945635, rs3761959, rs7522061, rs10489678 and rs7528684 SNPs in AR patients and healthy control individuals.

**Haplotype**	**AR (%)**	**Control(%)**	**x2**	**P**	**OddsRatio (95%CI)**
C A C G A	47.06(4.4)	51.73(4.3)	0.139	0.709	0.926[0.617~1.389]
C A C G G	33.39(3.1)	59.19(4.9)	6.995	8.19×10^-3^	0.560[0.363~0.865]
C A T G A	89.63(8.3)	91.34(7.6)	0.000	0.986	1.003[0.738~1.363]
C A T G G	38.32(3.5)	49.66(4.1)	1.289	0.256	0.779[0.506~1.200]
C G C G A	63.18(5.9)	77.13(6.4)	1.193	0.275	0.825[0.584~1.166]
C G C G G	34.25(3.2)	34.57(2.9)	0.003	0.959	1.013[0.626~1.639]
C G T G A	117.66(10.9)	77.79(6.5)	10.066	1.52×10^-3^	1.625[1.201~2.198]
C G T G G	39.40(3.6)	93.25(7.8)	22.309	2.36×10^-6^	0.406[0.277~0.597]
G A C G A	27.50(2.5)	43.62(3.6)	3.445	0.063	0.633[0.389~1.030]
G A C G G	39.99(3.7)	16.17(1.3)	10.953	9.40×10^-4^	2.596[1.446~4.659]
G A T G A	103.38(9.6)	58.86(4.9)	14.449	1.45×10^-4^	1.895[1.357~2.646]
G A T G G	38.85(3.6)	41.14(3.4)	0.027	0.869	0.963[0.615~1.508]
G G C G A	78.31(7.3)	37.37(3.1)	16.396	5.19×10^-5^	2.247[1.505~3.354]
G G C G G	13.44(1.2)	84.96(7.1)	53.283	3.0110^-13^	0.149[0.083~0.267]
G G T G A	110.32(10.2)	66.21(5.5)	13.243	2.76×10^-4^	1.798[1.306~2.474]
G G T G G	47.31(4.4)	59.01(4.9)	1.110	0.292	0.809[0.546~1.200]

AR, allergic rhinitis.

**Table 5:** Frequencies of the haplotypes formed by rs7528684, rs10489678 and rs7522061 SNPs in AR patients and healthy control individuals.

**Haplotype**	**AR (%)**	**Control(%)**	**x2**	**P**	**Odds Ratio(95%CI)**
A A C	30.67(2.8)	72.98(6.1)	13.767	0.209×10^-3^	0.451[0.294~0.694]
A A T	52.81(4.9)	81.50(6.8)	3.707	0.054	0.706[0.494~1.008]
A G C	216.10(20.0)	210.96(17.6)	2.204	0.138	1.173[0.950~1.448]
A G T	419.41(38.8)	291.56(24.3)	55.984	8.29×10^-14^	1.978[1.652~2.368]
G A C	30.57(2.8)	38.64(3.2)	0.292	0.589	0.876[0.541~1.418]
G A T	43.95(4.1)	64.89(5.4)	2.239	0.135	0.742[0.501~1.098]
G G C	121.65(11.3)	196.42(16.4)	12.336	4.48×10^-3^	0.649[0.509~0.827]
G G T	164.83(15.3)	243.06(20.3)	9.647	1.908×10^-3^	0.709[0.570~0.881]

AR, allergic rhinitis.

In short, the CGTGA, GATGA and GGTGA haplotypes were associated with a significantly increased risk of AR, while CGTGG and GGCGG were associated with a notably decreased risk of AR. Moreover, when the haplotypes of the three positive SNPs were analyzed, the haplotype of AGT increased significantly in AR cases, while GGC, GGT were associated with a markedly decreased risk of AR.

## Discussion

FCRL3 in human encodes a type I transmembrane protein harboring both cytoplasmic ITAM and ITIM elements that can repress B-cell receptor activation. Despite this inhibitory property, mounting associations for FCRL3 with autoimmune and lympho-proliferative disorders imply a role for it in promoting B-cell pathogenesis. High FCRL3 expression on B-cells and augmented autoantibody production were observed in individuals with the disease-susceptible genotype. Associations were also found between the SNP and susceptibility to autoimmune thyroid disease and systemic lupus erythematosus. Thus, FCRL3 may play a pivotal role in autoimmunity. As an important genetic autoimmunity factor, it functions as the mediator between the innate and adaptive immune system. AR, as an atopic desease, is also regulated by the innate and adaptive immune system, especially the imbalance of Th1/Th2 Cells. However, no public literature has revealed the association between polymorphisms of FCRL3 and AR until now. This study was performed to evaluate the potential association of FCRL3 polymorphisms with AR in a Chinese Han population.

In this study, we investigated the FCRL3 gene polymorphisms in a Chinese population with AR. The results demonstrated a significant association between SNPs rs7528684, rs10489678, rs7522061 in the FCRL3 gene and AR in a Chinese Han population. For rs7528684, which lies in the promoter region of FCRL3, the AA genotypes and the A allele significantly increased the risk of AR, but the AG, GG genotypes and the G allele decreased the risk of AR. Concerning rs10489678, located in the intron region of FCRL3, the GG genotype and the G allele were risk factors for AR, but the AG genotype and the A allele provided protection. For rs7522061, located in the extron4 region of FCRL3, the TT genotype and the T allele significantly increased the risk of AR, but the CT genotype and the C allele decreased the risk of AR. There is also a weak protective association between SNP rs945635 (in the exon2 region of FCRL3) and AR cases at the CC genotype and the G allele. However, the expression of SNP rs3761659 (in the intron region of FCRL3) shows no statistical difference between these two groups. Thus it can be seen that these SNPs in the promoter, extron and intron region of FCRL3 might contribute its functional effect on AR. According to the haplotypes analysis of the FCRL3 gene, we found that the CGTGA, GATGA and GGTGA haplotypes were associated with a significantly increased risk of AR, while CGTGG and GGCGG were associated with a notably decreased risk of AR. Concerning the three positive SNPs of FCRL3, the haplotype of AGT increased significantly in AR cases. To the best of our knowledge, this is the first study to evaluate an association between FCRL3 SNPs and AR in a Chinese Han population.

For SNPs rs945635 and rs3761959, there are a few inconsistent conclusions to be a risk factor of various autoimmune diseases. And these results are inconsistent between distinct ethnicities. A study [29] suggests the SNPs rs94563 and rs3761959 (except rs7528684) would not have a substantial effect in determining susceptibility to RA in populations of Northern European descent. Another studies show the genetic variations of SNPs rs945635 and rs3761959 in FCRL3 were not associated with SLE [30], but met the genome-wide association significance level (*P*c = 2.27×10^-12^ and 7.11×10^-13^, respectively) to Graves’ disease [31] in Chinese population. An updated meta-analysis [32] showed that significant associations were found to be correlated with autoimmunity diseases (ADs) risk for the SNP rs945635 in Europeans, North Americans and mixed group, and for rs3761959 in North Americans. In this study, we found a weak protective association between SNP rs945635 and AR cases at the CC genotypes and the G allele, and no statistical difference between these two groups for SNP rs3761659. Therefore, these two polymorphism loci would not be chosen as target SNPs of FCRL3 gene to investigate the genetic pathogenic process of the autoimmunity diseases, or AR.

For SNPs rs7528684, rs10489678, rs7522061 in FCRL3, this study found that there were significant association with AR in a Chinese Han population. The FCRL3 rs7528684 polymorphism has been shown to be a risk factor of various autoimmune diseases, including SLE, these results are inconsistent between distinct ethnicities. The updated meta-analysis [32] showed that the TC, TT + TC genotypes of rs7528684 contributed to a lower risk of ADs, compared with the CC carriers (OR = 0.91, 95% CI = 0.85–0.97; OR = 0.91, 95% CI = 0.85–0.98). In comparison with rs7528684 TC genotype, the TT + CC carriers were significantly associated with higher autoimmune diseases risk (OR = 1.03, 95% CI = 1.00–1.07). In terms of stratified analyses by ethnicity and disease phenotypes, there were significant associations of rs7528684 polymorphism both with ADs in Asians and Europeans, and with rheumatoid arthritis, Graves’ disease, type-1 diabetes, and other ADs under different genetic models. The FCRL3 (rs7528684) SNP has also been demonstrated a significantly increased frequency of the C/C genotype in Polish women with endometriosis-associated infertility than controls [33]. It alters the expression of FCRL3 and can be a risk factor of endometriosis-related infertility. Based on another study [34], it is not a risk factor of SLE in the Polish population, but this polymorphism may contribute to autoantibody production in this disease. Simmonds MJ and his colleague [35] have launched a study. It revealed that the significant association between the tag SNPs of FCRL3 (rs10489678, rs11264798) and GD, was prior to that with the FCRL5 in 5192 UK Caucasian cases and controls. Two SNPs, rs7528684 and rs7522061, in the FCRL3 gene differed notably between in 645 Caucasians MS patients and 786 controls, and also with high linkage disequilibrium; and the C allele of rs7528684 was found to be protective for MS [25]. A research [36] reveals no significant association the SNP rs7522061 in FCRL3 gene and susceptibility to HLA-B27-positive ankylosing spondylitis (AS) in 169 Chinese Han patients and 184 controls (HLA-B27-positive).

**Figure 1:**
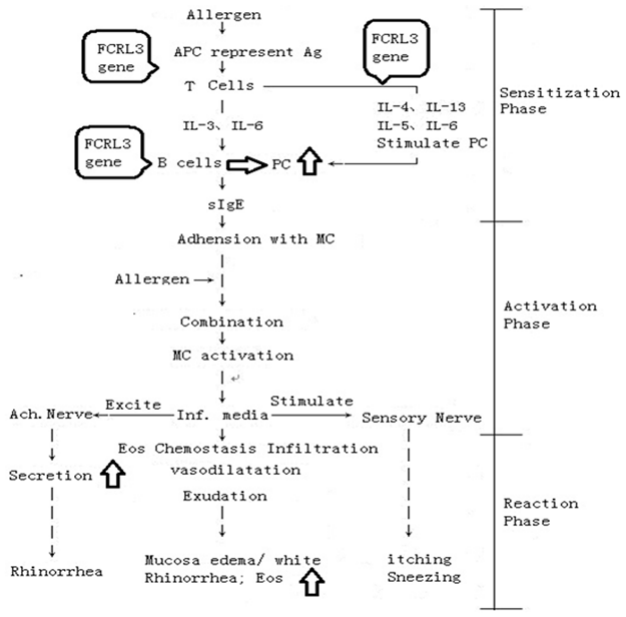
FCRL3 gene might be involved into the autoimmunity sensitization phase in the pathogenesis of AR. PC: plasma cell, MC: mast cell, Eos: eosinophilic granulocyte.

As discussed above, The SNPs in FCRL3 gene are strongly associated with ADs by way of mediating the pathogenesis of B cells, Th2/Th1 Cells and Treg Cells, while these cells involve similarly into AR. As shown in Fig. 1, the pathogenesis of B cells, Th2/Th1 Cells and Treg Cells triggered by Allergen play the dominant role in the sensitization phase of AR. Therefore, the SNPs in FCRL3 might involve into this process by way of mediating these effective cells. In other words, the Polymorphisms in FCRL3 could play an important autoimmunity role in AR pathogenesis by mediating functions of B/T Cells. However, the classic pathogenic theory stress on that AR mainly caused by the Th1/Th2 imbalanced IgE-mediated Type I allergic reaction. The dominant Th2 cells undertake the key task to trigger subsequent reaction at the first sensitization phase of AR. Th1 cells mainly are associate with ADs, but not with AR. Our study suggests that the autoimmunity might also play a key role in the pathogenesis of AR. FCRL3 gene might just be the potential risk intervened with this pathogenic process of AR, though its involving path keeps under clear.

In short, our findings that the SNPs rs7528684, rs10489678, rs7522061 in FCRL3 gene, which revealed in the strong association with Chinese Han AR patients, have been testified by a few researches focused on their association with numerous ADs. Moreover, the haplotype analysis reveals that the haplotype of AGT in the FCRL3 gene increased significantly in AR cases. Thus, we postulated that the SNPs, rs7528684, rs10489678 and rs7522061 might be chosen as the tag SNPs to evaluate the high risk of developing AR in Chinese Han population.

In the present study, we made many efforts to decrease the influence of confounding factors on the results. We selected the AR patients and controls using strict guidelines and confirmed the genotyping results by direct sequencing. We observed a novel association between rs945635, rs3761959, rs7522061, rs10489678 and rs7528684 of FCRL3 and AR in a Chinese Han population. However, it is worth mentioning that there are some limitations to our study. First, we did not detect the protein level of FCRL3 in the peripheral blood and perform a functional analysis study, so we could not draw a certain conclusion about the influence of these polymorphisms on the cytokine levels. Second, as it is well known that environmental factors are critical for the development of AR, further studies will be needed to clarify the genetic influence of FCRL3 in AR pathogenesis, including gene-gene and gene-environment interactions.

In summary, we have demonstrated an association between FCRL3 gene polymorphisms and susceptibility to AR. Our results suggest that there may be an autoimmunity player evolved into the pathogenic process of AR. The FCRL3 are susceptibility genes for the autoimmunity development of AR in the Chinese Han population. However, more intensive studies and advanced methods, such as genome-wide association study, gene-gene, gene-environment, are required to further clarify the molecular mechanisms and the complex interaction between environmental factors and FCRL3 gene polymorphisms in AR. Generally, we hope that our study can provide a deeper insight into AR pathogenesis.
